# Foliar Application of a New Biostimulant at Key Growth Stages Improves Soybean Performance

**DOI:** 10.3390/plants15101519

**Published:** 2026-05-15

**Authors:** Luiz Gustavo Moretti, João William Bossolani, José Roberto Portugal, Tatiani Mayara Galeriani, Francesco Magro, Eleonora Perucco, Giacomo Masetti, Carlos Alexandre Costa Crusciol

**Affiliations:** 1 Embrapa Cerrados, Planaltina 73310-970, Brazil; luiz.moretti@embrapa.br; 2 School of Agricultural Sciences (FCA), São Paulo State University (UNESP), Botucatu 18610-034, Brazil; bossolani.agro@gmail.com (J.W.B.); jose.portugal@unesp.br (J.R.P.); tatiani.galeriani@unesp.br (T.M.G.); 3 Sofbey SA by Sipcam Oxon, CH-6850 Mendrisio, Switzerland; fmagro@sipcam.com (F.M.); eperucco@sofbey.com (E.P.); gmasetti@sofbey.com (G.M.)

**Keywords:** biostimulants, plant resilience, physiological stimulus, abiotic stress

## Abstract

Soybean is one of the most important crops worldwide, but its productivity is frequently challenged by abiotic stresses such as drought and heat, which impair physiological and metabolic processes. Biostimulants have emerged as sustainable tools to improve plant performance under adverse conditions. This study evaluated the effects of foliar application of a new biostimulant, “SB”, on soybean photosynthetic efficiency, antioxidant metabolism, biometric traits, and grain yield. SB was applied at different doses (0.5, 1.0, 1.5, and 2.0 L ha^−1^) at the V_4_ and R_1_ growth stages during two seasons (2023/2024 and 2024/2025). Foliar SB application enhanced soybean leaf chlorophyll levels, RuBisCO activity, and gas exchange parameters, resulting in higher photosynthetic rates, carboxylation efficiency, and water use efficiency. In addition, foliar SB application reduced hydrogen peroxide and malondialdehyde accumulation, indicating lower oxidative damage and improved redox balance. These physiological and metabolic improvements contributed to greater root development and plant height and significant increases in yield components. Grain yield was consistently improved by all SB application rates, but the 1.5 L ha^−1^ dose produced the most stable and positive effects across both seasons, with an average increase of more than 500 kg ha^−1^ compared to the control. Overall, foliar SB application proved to be an efficient and promising management strategy to enhance soybean resilience and productivity under variable climatic conditions.

## 1. Introduction

Soybean is the leading agricultural crop in Brazil, the world’s largest producer of this commodity [1], and has a high export rate due to its significant value in the international grain market. Global demand for soybean (*Glycine max* (L.) Merrill) is driven by the production of plant-based proteins and oils for human consumption and use as animal feed [2,3,4]. However, rapid and ongoing climate change has exacerbated the vulnerability of soybean productivity to environmental stresses, particularly abiotic stresses linked to climatic factors [5]. In Brazil, demand for soybean has fueled the expansion of cultivation into new agricultural frontiers, often in regions where the soil and climatic conditions present additional challenges for production [2,3,4].

Abiotic stresses encompass physical and chemical phenomena such as drought, salinity, flooding, heavy metal contamination, and extreme temperatures, all of which adversely impact the physiological and metabolic processes of plants, hindering their growth, development, and grain production [5,6,7]. The abiotic stress processes commonly observed in the field also trigger oxidative stress, an internal metabolic response. The excessive buildup of reactive oxygen species (ROS) damages plant cells by promoting lipid peroxidation in membranes [8,9,10,11]. If not neutralized, ROS can disrupt plant development, leading to leaf abscission, changes in root gravitropism, and damage to the photosynthetic pathway, all of which directly affect crop productivity [3,12,13].

To minimize the damage caused by plant exposure to adverse environmental conditions, new technologies are being developed that target both crop physiology and field management [4]. In particular, biostimulants derived from natural raw materials offer an innovative approach to modify or regulate plant physiological processes to promote growth, mitigate stress effects, and enhance productivity [8,14,15]. These raw materials include extracts from algae, humic and fulvic acids, amino acids and peptides, beneficial microorganisms, plant hormones, polysaccharides, phenolic compounds, and even chitosan, which is derived from crustacean exoskeletons [15,16,17,18,19]. These resources are highly valued in agriculture for their contents of macro- and micronutrients, biopolymers, polysaccharides, vitamins, amino acids (both proteogenic and non-proteogenic), polyamines, and other molecules that support and enhance plant metabolism [20]. Biostimulants have been reported to enhance plant physiology by promoting nutrient absorption and utilization, boosting resistance to stress, and activating antioxidant defense systems. This activation plays a key role in controlling ROS accumulation and protecting the photosynthetic apparatus under stress conditions. In addition, biostimulants can stimulate the production of metabolic compounds and defense-related responses, contributing to improved plant performance and yield stability under adverse conditions [6,21,22,23,24,25,26,27,28].

Despite these advances, there is still limited information on the physiological and biochemical mechanism underlying the action of novel biostimulant formulations under field conditions, particularly those based on complex molecular structures. In addition, integrated evaluations combining antioxidant metabolism and photosynthetic performance remain poorly explored, limiting a more comprehensive understanding of their mode of action [29].

Advancing the development of biostimulants for soybean production requires a deep understanding not only of soybean cultivation but also of other agricultural species and their responses and adaptations to climate change. Equally important is understanding the impacts of various types of biostimulants on these responses and productivity. Stress factors should not obstruct the growing global demand for food, the need for increased crop productivity, and the enhancement of plant resilience in environments vulnerable to adverse climatic conditions. This study represents a pre-market validation aimed at investigating the effects of different dose rates of a promising novel biostimulant (“SB”, hereinafter) on soybean plants. Currently under development by Sofbey SA, Mendrisio, Switzerland, “SB” formulation is based on the Glicoligno Lipidic Complex, obtained through a company-developed methodology. Glicoligno Lipidic Complex can be defined as a mixture comprising an aliphatic lipidic fraction, a carbohydrate fraction, and an aromatic fraction, which were characterized by NMR. The main objective of this investigation is to assess the biostimulant impact of foliar “SB” application on antioxidant metabolism, photosynthetic performance, agronomic traits, and soybean grain yield.

## 2. Results

### 2.1. Leaf Photosynthetic Pigment Content and RuBisCO Activity

Foliar application of SB had positive effects on leaf photosynthetic pigment content and RuBisCO activity (Table 1). Significant effects on chlorophyll *a* content were observed only in the 2024/2025 season, where application Time A significantly increased chlorophyll *a* content at 14 DAA, with higher concentrations at higher doses. This initial effect persisted at 7 and 14 DAB. At 14 DAB, a 10% increase in chlorophyll *a* content was recorded in SB 2.0 compared with the control, with no significant difference between SB 2.0, SB 1.0 and SB 1.5. For chlorophyll *b*, effects of foliar SB application were evident only at 14 DAA in both seasons. SB 1.0 increased chlorophyll *b* content by 7.6% and 9.6% compared to the control in 2023/2024 and 2024/2025, respectively, with no significant difference compared to SB 1.5 (+4.8% and +8.5%) and SB 2.0 (+4.0 and +10%). Total chlorophyll increased at 14 DAA in both seasons and at 7 and 14 DAB only in 2024/2025. The highest increase was observed at 14 DAB in 2024/2025 (+9.6%), with no significant differences among SB treatments. Carotenoid content showed a slight increase at 7 DAB in 2024/2025 (+6.1%), also without differences among doses.

RuBisCO activity was significantly influenced by both SB applications, and these increases persisted at 14 DAB. RuBisCO activity was highest in SB 2.0, with increases of 12% and 10% compared to the control in 2023/2024 and 2024/2025, respectively, but did not differ significantly between SB 2.0, SB 1.0 (+15% and 7.9%), and SB 1.5 (+12% and 8.6%).

### 2.2. Gas Exchange Parameters

In general, gas exchange parameters were influenced by SB application (Table 2). Significant increases in the net photosynthetic rate (*A*) were observed after both applications (except after application A in 2023/2024), particularly at intermediate and high doses, with no significant differences among SB treatments. Increases in stomatal conductance (*gs*) were limited and observed only at 14 DAB in 2023/2024, without differences among doses. The internal CO_2_ concentration (C_i_) decreased under SB application, indicating greater CO_2_ assimilation, with consistent effects across evaluation periods. Leaf transpiration (E) showed slight reductions, while water use efficiency (WUE) increased across doses, with a persistent effect up to 14 DAB. Positive effects of SB application on carboxylation efficiency (given by the *A*/*Ci* ratio) were also enhanced, especially at intermediate and high doses, with no significant differences among SB treatments.

Overall, the combined responses of A, Ci, WUE, and carboxylation efficiency indicate a more efficient CO_2_ assimilation and water use under SB application. These results indicate that the positive effects of foliar SB application were maintained even after two weeks, with no significant differences among doses, although intermediate doses (1.0–1.5 L ha^−1^) tended to show more consistent responses.

### 2.3. Oxidative Stress and Antioxidant Metabolism

Foliar SB application alleviated oxidative stress, as evidenced by decreases in ROS levels and antioxidant enzyme activity (Table 3). SB application reduced leaf hydrogen peroxide (H_2_O_2_) content at 7 and 14 DAA in both seasons and at 7 and 14 DAB in 2024/2025. These effects were maintained over time, and at 14 DAB in 2024/2025, H_2_O_2_ content was reduced by 7.1% in SB 1.5 and 9.6% in SB 2.0 compared to the control. Similarly, lipid peroxidation, estimated by malondialdehyde (MDA) content, was reduced by SB 1.0 (−21.6%) in 2024/2025 and SB 1.5 (−18.5% and −16.3%) and SB 2.0 (−15.8% and −17.4%) in 2023/2024 and 2024/2025, respectively. The reductions in H_2_O_2_ and MDA levels were accompanied by decreases in the activities of superoxide dismutase (SOD) and catalase (CAT) (Table 3). Reductions in SOD activity were observed at 7 DAA and 7 DAB in both seasons, at 14 DAA in 2024/2025, and at 14 DAB in 2023/2024. At 14 DAB in 2023/2024, SOD activity was reduced by 7.4% and 5.4% in SB 1.5 and SB 2.0, respectively, compared to the control. CAT activity was reduced at 7 DAA in both seasons and at 7 and 14 DAB in 2023/2024. At 14 DAB in 2023/2024, SB 2.0, SB 1.5, SB 1.0, and SB 0.5 reduced CAT activity by 40%, 39%, 32% and 19%, respectively, compared to the control.

### 2.4. Biometric Parameters and Grain Yield at Harvest

The increases in photosynthetic pigment levels and reductions in oxidative stress produced by foliar SB application were reflected in significant gains in root dry weight (RDW), plant height (PH), grain yield and yield components (Table 4). Overall, SB application promoted consistent improvements across seasons, particularly at intermediate and high doses. Compared with the control, RDW increased by 11% in SB 1.5 and SB 2.0 and by 7.7% in SB 1.0 in 2023/2024. In 2024/2025, PH increased by 6.3%, 9.5%, and 12% in SB 1.0, SB 1.5, and SB 2.0, respectively, compared to the control. The number of grains per pod (NGP) was 2.7% and 4.0% higher in SB 1.5 and SB 2.0, respectively, in 2024/2025. The number of grains per plant increased by 8.1% in SB 1.0 in 2024/2025, by 12% and 9.7% in SB 1.5 in 2023/2024 and 2024/2025, and by 8.4% and 7.3% in SB 2.0 in 2023/2024 and 2024/2025. Grain yield also increased, with gains of 11% and 9.2% in SB 1.0 (+482 and +373 kg ha^−1^), 12% and 10% in SB 1.5 (+514 and +405 kg ha^−1^), and 7.9% and 11% in SB 2.0 (348 and 464 kg ha^−1^) in 2023/2024 and 2024/2025 compared to the control.

Grain yield gains ranged from approximately 8% to 12%, corresponding to an increase of 514 kg ha^−1^ compared to the control. These gains are relevant, indicating that SB application can contribute to meaningful improvements in crop productivity under field conditions. Although no significant differences were observed among doses, the 1.5 L ha^−1^ rate tended to provide more stable responses across seasons.

## 3. Discussion

Soybean production is a pillar of the Brazilian economy but is threatened by increasing climate challenges [30,31,32]. Between the two seasons, rainfall was more irregular in 2023/2024. In that season, water deficits coincided with the treatment applications and temperatures frequently exceeding 35 °C, creating a combined drought and heat stress scenario that negatively affected plant growth and development. Conditions were more favorable for crop development in 2024/2025, with better distributed rainfall and more stable temperatures. Although the accumulated rainfall was similar between the two seasons (709 mm in 2023/2024 and 720 mm in 2024/2025), its distribution was decisive: in 2023/2024, lower rainfall volumes during the vegetative growth period up to flowering (V2 to R1 stages) reduced soybean performance, whereas in 2024/2025, water availability was sufficient for proper crop establishment. Soybean water demand varies from 450 to 800 mm throughout the season, with higher sensitivity in the flowering and grain filling stages, highlighting the importance of adequate rainfall distribution for productivity [33].

Studies have shown that foliar application of biostimulants can benefit plant physiological and metabolic processes [34] and enhance their tolerance to abiotic stress [35,36]. In the present study, foliar SB application increased RDW, suggesting stimulation of root growth, which favors nutrient accessibility and uptake [37]. Corroborating these results, the application of humic and fulvic acids has been shown to significantly promote root growth and branching [38,39].

Foliar SB application increased chlorophyll levels and RuBisCO activity, resulting in higher photosynthetic efficiency. The increases in chlorophyll levels may be associated with improved nutrient availability, particularly nitrogen and magnesium, which are directly involved in chlorophyll biosynthesis and the structural stability of the photosynthetic apparatus [17,40]. Enhanced pigment content under biostimulant application has been linked to improved nutrient uptake [41]. Similarly, the application of a biostimulant based on algae extract increased photosynthetic pigment levels in rehydrated plants, favoring the use of light energy and contributing to recovery from water stress [42,43]. The higher RuBisCO activity, a key enzyme for CO_2_ fixation [44], may reflect improved nutritional status, especially the availability of nutrients required for enzyme synthesis and function [45]. Biostimulant application may help preserve photosynthetic metabolism under stress conditions, thereby supporting carbon assimilation and the maintenance of physiological activity [40].

The apparent physiological efficiency of soybean increased under foliar application of SB, as evidenced by the maintenance of C-assimilation while balancing C-gain and reduced water loss. Under stress conditions typically observed in the field, plants restrict gas exchange to limit transpiration, but this response also reduces CO_2_ availability in leaves and impairs photosynthetic performance [46,47]. Therefore, the combination of higher net photosynthesis and lower transpiration suggests that the biostimulant helped sustain carbon assimilation while improving WUE. The reduction in the internal CO_2_ concentration further supports the interpretation of more effective CO_2_ assimilation and greater carboxylation efficiency [4]. Our observations are consistent with previous reports that biostimulant application attenuates the physiological limitations imposed by drought by preserving photosynthetic activity and supporting metabolic adjustment [4,40]. Although positive effects of biostimulants on photosynthesis under stress have been reported frequently in the literature, transpiration responses may vary depending on the composition of the product, application strategy, and environmental conditions [40,48,49].

The maintenance of photosynthetic performance under stress may also be associated with lower oxidative stress, since a more stable photosynthetic metabolism tends to reduce ROS generation [4,50,51]. Foliar biostimulant application also contributed to strengthening plant antioxidant defenses. ROS are normal byproducts of plant metabolism and act as signaling molecules regulating gene expression; however, under stress conditions, excessive ROS accumulation can cause oxidative damage [40,52]. In the present study, foliar SB application decreased H_2_O_2_ and MDA levels and SOD and CAT activities, indicating reduced oxidative stress. This response can be explained as a consequence of a lower ROS generating pressure rather than a suppression of the antioxidant system. Antioxidant enzymes are strongly inducible, and their activity is primarily regulated by ROS availability. Thus, when ROS production is reduced, the metabolic demand for enzymatic detoxification also decreases. This effect may be related to higher photosynthetic efficiency and improved nutrient use, resulting in lower ROS generation, the maintenance of redox balance, and, consequently, a reduced need for antioxidant enzyme activation. This reduction in oxidative stress may be linked to a lower excitation pressure on photosynthetic apparatus, reducing electron leakage from the photosynthetic electron transport chain, which is a major source of ROS under stress conditions. As a result, the demand for antioxidation enzyme activity is reduced, reflecting a more stable redox environment rather than as an induced stress response [53]. In this context, the decrease in antioxidant enzyme activity does not indicate a weakening of the plant defense system. Instead, it reflects that the antioxidant metabolism is operating under a lower oxidative demand, maintaining redox homeostasis with reduced metabolism cost. Importantly, the simultaneous reduction in H_2_O_2_ and MDA levels indicates that oxidative damage was minimized, supporting that the antioxidant system remains functional and effective. In addition, although not directly measured, the reduction in oxidative stress indicators may be associated with the physicochemical properties of the biostimulant. Cuticular permeability depends on compound polarity and lipophilicity, suggesting that the lipidic fraction may enhance the delivery of glyco-lipid-derived compounds, thereby improving metabolic signaling and antioxidant regulation [17,54]. In contrast to reports that fulvic acids increase antioxidant enzyme activity in maize [54] and wheat [55], our results suggest that SB application effectively reduced oxidative stress by preventing ROS formation upstream, highlighting an additional mechanism by which the biostimulant confers antioxidative benefits. Furthermore, normalization of photosynthetic rates contributes to reducing cellular damage by redirecting free electrons that would otherwise form free radicals [54,56].

Foliar application of the biostimulant improved plant metabolism, resulting in increases in yield components, including grains per plant, and grain yield. These increases were associated with improvements in the root system, photosynthetic capacity, WUE, and antioxidant metabolism. Notably, the responses observed across physiological and biochemical parameters indicate the intermediate dose (1.5 L ha^−1^) was sufficient to achieve maximum plant performance, with no additional gains at the highest dose. This suggests a moderate concentration optimizes metabolic processes, while higher doses do not result in proportional increases. This behavior may be associated with the attainment of a functional metabolic threshold, beyond which additional inputs do not enhance efficiency and may increase the metabolic cost of maintaining cellular homeostasis.

## 4. Materials and Methods

### 4.1. Experimental Area Description

The soybean (*Glycine max* (L.) Merrill) field trials were conducted over two growing seasons: 2023/2024 and 2024/2025. The experiments were carried out at the Lageado Experimental Farm, College of Agricultural Sciences (UNESP/FCA), Botucatu, Sao Paulo State, Brazil (22°49′30.0″ S 48°25′40.7″ W), during the period from November to March in both seasons, under no-tillage management. The experimental area has an average elevation of 792 m, and the soil was classified with chemical characterization performed prior to the installation of the trials. For soil analysis, 10 subsamples were randomly collected within the experimental area and combined into a single composite sample. The soil classification and chemical characterization results are presented in Table 5.

**Figure 1:**
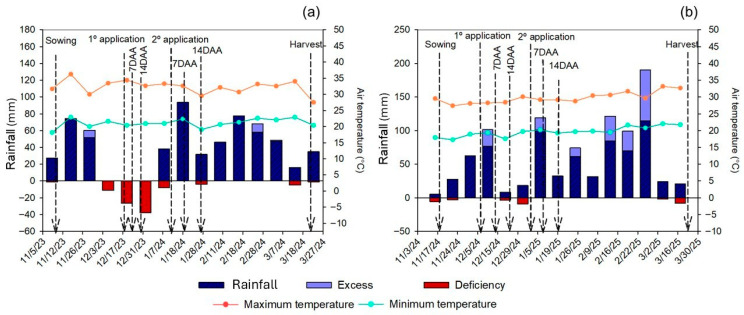
Rainfall and maximum and minimum temperatures during the experimental period: (**a**) 2023/2024 season; (**b**) 2024/2025 season.

According to the Köppen–Geiger classification system, the climate in the region is characterized as tropical savanna (Aw), with an average annual temperature of 21.3 °C and average annual precipitation of approximately 1500 mm. The tendency for short dry spells in Central–West Brazil during the monsoon season (from October to April) was comprehensively established by an analysis of a historical series of 37 austral summer seasons (from 1979 to 2016) [2]. Because weather conditions can influence crop development and product effectiveness, meteorological data (rainfall, solar radiation, wind speed, relative humidity, and maximum and minimum temperatures) were recorded by an automatic meteorology station installed near the experimental area from seven days before the experiment began until seven days after the final evaluation. The evapotranspiration reference (ET_0_) was calculated based on the Penman–Monteith methodology [3]. Soybean evapotranspiration (ETc) was calculated using the crop coefficient (Kc) for each phenological stage [3]. Rainfall data were used to monitor the climatological water balance, which was calculated using an electronic spreadsheet [4] following the procedure to determine real evapotranspiration (ETr) [5]. The measured parameters, including precipitation, humidity, and temperature, are shown in Figure 1.

The soybean variety NEO610 IPRO was used in both seasons. This variety is known for its high yield potential and has a medium growth cycle of 109 days. It is highly adaptable to different soil types and exhibits resistance to diseases such as stem canker, frogeye leaf spot, and phytophthora root rot. Although recommended for high-fertility soils, it also performs well in lowland areas and under off-season planting conditions.

### 4.2. Experimental Design and Treatments

The experimental area followed a randomized complete block design (RCBD) consisting of five treatments with four replications each. The number of replications was determined to ensure a minimum of 10 residual degrees of freedom. In total, 20 experimental plots were established, each measuring 63 m^2^, with a row spacing of 0.45 m. Additional crop management information is provided in Table 6.

The treatments were as follows: (1) control—water application only; (2) application of the biostimulant SB at 0.5 L ha^−1^; (3) SB at 1.0 L ha^−1^; (4) SB at 1.5 L ha^−1^; (5) SB at 2.0 L ha^−1^. Applications were carried out at two soybean phenological stages: Time A—*V*_4_ *vegetative stage* (four fully developed trifoliate leaves) and Time B—*R*_1_ *reproductive stage* (beginning bloom—first flower at any node on the main stem) (Table 7).

Product applications were performed using a CO_2_-pressurized backpack sprayer equipped with a 3 m boom containing six flat-fan nozzles (AXI 11002) spaced 0.50 m apart. Spray volume and pressure were set at 150 L ha^−1^ and 1.80 bar, respectively.

### 4.3. Root Development (Root Dry Weight)

At the full vegetative development stage, root samples were collected to quantify root biomass. Sampling was performed using a cylindrical steel auger designed to reach the 0–0.20 m soil layer along the planting row. The procedure was carried out carefully to avoid loss or damage to root structures. Roots from three representative plants per plot were sampled, pooled, and gently washed with running water over a fine mesh sieve to remove all adhering soil without harming the finer root structures. After cleaning, the samples were dried in a forced-air oven at 65 ± 2 °C until a constant weight was achieved. Dry weight was then determined using an analytical balance with 0.001 g precision. The results were expressed in grams per plant (g plant^−1^), using the average of the three plants per plot for subsequent statistical analysis.

### 4.4. Leaf Analyses

**Figure 2:**
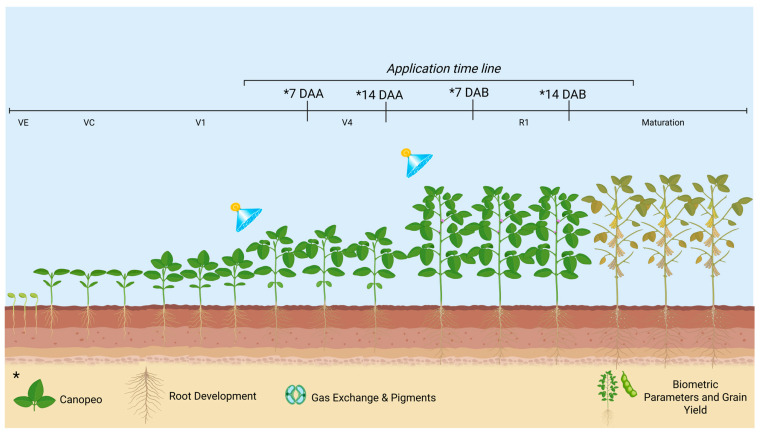
Graphical illustration of the experimental timeline in each growing season, including timing of biostimulant application and sampling to determine effects on antioxidant metabolism, photosynthetic and agronomic parameters, and soybean grain yield. * Represents the type of evaluation performed at the respective phenological stage.

Leaf, physiological, and enzymatic analyses were conducted at 7 and 14 days after application A (DAA) and 7 and 14 days after application B (DAB), corresponding to the V_4_ (fourth fully developed trifoliate leaf; Time A) and R_1_ (beginning of flowering; Time B) growth stages, respectively. The specific sampling dates are presented in Table 2 and Figure 2. For both laboratory analyses and gas exchange measurements, the third fully expanded leaf from the apex of the central stem was used. Five plants per plot were evaluated for each measurement.

### 4.5. Leaf Photosynthetic Pigment Analyses

Photosynthetic pigments, including chlorophyll a, chlorophyll b, total carotenoids, and total chlorophylls, were analyzed using leaf discs (0.5 cm in diameter) taken from the lamina, between the leaf margin and the central vein. The discs and 2 mL of N,N-dimethylformamide (DMF) were placed in Eppendorf tubes wrapped in aluminum foil to prevent light exposure and incubated in the dark for 24 h [57]. After the extraction period, pigment concentrations were quantified by spectrophotometry at wavelengths of 664 nm for chlorophyll a, 647 nm for chlorophyll b, and 480 nm for total carotenoids [58].

### 4.6. Gas Exchange Analyses

The net photosynthetic rate (A, μmol m^−2^ s^−1^), stomatal conductance (Gs, mol m^−2^ s^−1^), intercellular CO_2_ concentration (Ci, μmol mol^−1^), transpiration rate (E, mmol m^−2^ s^−1^), carboxylation efficiency (EC), and water use efficiency (WUE) were measured using an infrared gas analyzer (IRGA, model CIRAS-3, PP Systems). Measurements were conducted on five randomly selected plants per plot during the morning hours between 9:00 and 11:00 a.m. with a controlled ambient CO_2_ concentration of 390 μmol mol^−1^.

### 4.7. Oxidative Stress and Antioxidant Metabolism

For enzymatic activity evaluations, the sampled leaf tissue was quickly frozen in liquid nitrogen and stored at −80 °C in Falcon tubes. To prepare the leaf extract, approximately 500 mg of plant tissue was ground in liquid nitrogen with 10% (*v*/*v*) polyvinylpolypyrrolidone (PVPP). The resulting homogenate was mixed with 100 mM potassium phosphate buffer (pH 7.5) at a 1:3 ratio. The buffer was prepared using K_2_HPO_4_ and KH_2_PO_4_ and supplemented with 3 mM dithiothreitol (DTT) and 1 mM ethylenediaminetetraacetic acid (EDTA) [58]. The suspension was centrifuged at 5000 rpm for 10 min, and the supernatant was collected as the crude enzymatic extract. Total protein content in the extract was determined using the Bradford method [59] prior to enzymatic assays. For the Bradford assay, 100 µL of the extract was mixed with 5 mL of Bradford reagent, and the absorbance at 595 nm was recorded after a 15 min incubation.

Lipid peroxidation was assessed by measuring malondialdehyde (MDA) levels, determined by the reaction of MDA with thiobarbituric acid (TBA) [60]. The absorbance of the MDA–TBA complex at 532 nm was determined using a spectrophotometer. The MDA concentration was calculated by referring to a standard curve prepared with 1,1,3,3-tetramethoxypropane (TPE) and an extinction coefficient of 155 mM^−1^ L^−1^. The results were reported as nanomoles of MDA per gram of fresh weight (nmol g^−1^ FW). Hydrogen peroxide (H_2_O_2_) levels were quantified based on a calibration curve and expressed in mol g^−1^ FW [61].

Superoxide dismutase (SOD) activity was evaluated using a photochemical assay containing 13 mM methionine, 100 nM EDTA, 2 µM riboflavin, and 75 µM nitroblue tetrazolium (NBT) in 50 mM potassium phosphate buffer [62]. SOD activity was expressed in units per gram of protein (U g^−1^ protein). Catalase (CAT) activity was determined by monitoring the decomposition of hydrogen peroxide (250 µM) and expressed as nanomoles of H_2_O_2_ consumed per minute per milligram of protein (nmol min^−1^ mg^−1^ protein) [63,64].

### 4.8. Biometric Parameters and Grain Yield at Harvest

At soybean physiological maturity, 10 consecutive plants per plot were evaluated to determine plant height, number of pods per plant, number of grains per pod, and number of grains per plant. The weight of 100 grains adjusted to 13% moisture (wet basis) and grain yield were measured by harvesting the experimental useful area.

### 4.9. Statistical Analysis

The data were first subjected to error normality analysis [65] and homoscedasticity of variances [66]. Data meeting these assumptions were subjected to analysis of variance (ANOVA) with an F-test applied at a probability level of *p* ≤ 0.10, and means were compared using Fisher’s protected *t*-test at *p* ≤ 0.10.

## 5. Conclusions

The results of this study demonstrate that the foliar application of the new biostimulant “SB” is a viable and promising strategy to improve the physiological performance of soybean, particularly when applied at critical growth stages and at intermediate and high doses. The dose of 1.5 L ha^−1^ produced the most consistent results across both growing seasons, with enhancements in photosynthetic metabolism, water use efficiency, and antioxidant balance that translated into increases in yield components and grain yield, although not significantly different from the 1.0 L ha^−1^. Thus, foliar biostimulant application can serve as a complement to traditional soybean management practices, for promoting more resilient and sustainable production systems, especially under abiotic stress conditions, by improving physiological performance and contributing to reduced oxidative stress. In this context, the product offers an alternative strategy based on the modulation of plant physiological responses. These findings suggest that the biostimulant may contribute to improving crop performance under variable environmental conditions. Future research should focus on long-term evaluations across different scenarios and elucidating the biochemical and molecular mechanisms involved.

## Figures and Tables

**Table 1:** Concentrations of photosynthetic pigments (chlorophyll *a*—Chl *a*; chlorophyll *b*—Chl *b*; total chlorophyll—Chl t; and total carotenoids—Car t) and RuBisCO activity in soybean leaves as a function of foliar SB application during the 2023/2024 and 2024/2025 growing seasons. DAA—days after application A; DAB—days after application B.

Treatment	Chl *a*		Chl *b*		Chl *t*		Car *t*		RuBisCO	
	mg g^−1^ Fresh Weight								µmol CO_2_ g^−1^ DW h^−1^	
	2023/24	2024/25	2023/24	2024/25	2023/24	2024/25	2023/24	2024/25	2023/24	2024/25
**Time A**										
**7 DAA at the V_4_ Phenological Stage**										
Absolute Control	1653	1808	497	409	2151	2216	433	442	123 c **	150 b
SB 0.5 L	1694	1941	509	425	2203	2366	442	436	138 b	154 b
SB 1.0 L	1673	1979	503	436	2176	2414	443	453	149 ab	166 a
SB 1.5 L	1669	1940	502	419	2171	2359	448	443	151 a	165 a
SB 2.0 L	1675	1992	504	425	2179	2418	443	451	146 ab	164 a
*p*-value	0.6491	0.2284	0.6248	0.8597	0.6519	0.1077	0.4481	0.9590	0.0100 *	0.0266 *
C.V. (%)	2.18	5.92	2.13	8.10	2.17	4.48	2.43	8.01	6.91	4.43
	**14 DAA at the V_4_ Phenological Stage**									
Absolute Control	1760	1545 b	514 c	477 b	2274 c	2022 b	468	442	117	149 c
SB 0.5 L	1762	1582 b	530 bc	518 a	2292 bc	2100 b	466	445	121	150 bc
SB 1.0 L	1831	1749 a	551 a	523 a	2381 a	2272 a	477	445	122	154 bc
SB 1.5 L	1833	1683 a	551 a	511 a	2384 a	2194 a	485	450	127	157 ab
SB 2.0 L	1819	1707 a	547 ab	526 a	2366 ab	2233 a	481	452	128	164 a
*p*-value	0.1451	0.0028 *	0.0142 *	0.0591 *	0.0879 *	0.0010 *	0.1900	0.9610	0.5407	0.0205 *
C.V. (%)	2.84	3.80	2.71	4.44	2.77	3.03	2.61	4.78	8.89	3.86
**Time B**										
	**7 DAB at the R_1_ Phenological Stage**									
Absolute Control	1632	1465 c	506	473	2138	1937 c	445	412 b	106 b	123 b
SB 0.5 L	1711	1520 bc	509	470	2221	1990 bc	449	430 a	106 b	134 a
SB 1.0 L	1725	1542 abc	519	497	2244	2040 ab	457	430 a	108 b	135 a
SB 1.5 L	1703	1583 ab	512	454	2216	2036 ab	451	437 a	123 a	137 a
SB 2.0 L	1725	1612 a	519	502	2243	2114 a	457	433 a	121 a	137 a
*p*-value	0.1339	0.0811 *	0.5746	0.1143	0.1406	0.0706 *	0.4905	0.0726 *	0.0102 *	0.0416 *
C.V. (%)	3.09	4.49	2.57	5.50	2.70	3.82	2.32	2.61	6.37	4.60
	**14 DAB at the R_1_ Phenological Stage**									
Absolute Control	1766	1592 c	509	451	2204	2051 c	437	431	133 b	140 c
SB 0.5 L	1727	1659 b	519	467	2246	2138 b	445	436	144 ab	145 b
SB 1.0 L	1705	1736 a	522	482	2227	2230 a	451	442	153 a	151 a
SB 1.5 L	1693	1722 a	526	457	2292	2188 ab	448	442	149 a	152 a
SB 2.0 L	1717	1755 a	516	480	2233	2247 a	454	445	149 a	154 a
*p*-value	0.3870	0.0023 *	0.6879	0.2509	0.2169	0.0016 *	0.6463	0.7051	0.0768 *	0.0004 *
C.V. (%)	3.08	2.82	3.38	4.76	2.26	2.46	3.67	3.36	6.48	2.24

* Data were analyzed by analysis of variance (ANOVA), and when significant differences were identified, means were compared using Fisher’s protected least significant difference (LSD) test at *p* < 0.10. ** Columns with different letters are significantly different according to the LSD test (*p* < 0.10).

**Table 2:** Net photosynthetic rate (*A*—µmol CO_2_ m^−2^ s^−1^), stomatal conductance (*gs*—mol H_2_O m^−2^ s^−1^), intercellular CO_2_ concentration (*Ci*—µmol CO_2_ mol^−1^), leaf transpiration rate (*E*—mmol H_2_O m^−2^ s^−1^), water use efficiency (WUE—μmol CO_2_ (mmol H_2_O)^−1^), and carboxylation efficiency (CE—dimensionless) in soybean diagnostic leaves as a function of foliar SB application during the 2023/24 and 2024/25 growing seasons.

Treatment	*A*		*Gs*		*Ci*		*E*		WUE		CE	
	2023/24	2024/25	2023/24	2024/25	2023/24	2024/25	2023/24	2024/25	2023/24	2024/25	2023/24	2024/25
**Time A**												
**7 DAA at the V_4_ Phenological Stage**												
Absolute Control	21.4	23.7 b **	224	247	194 a	176 a	6.21 a	4.15	3.45 b	5.75	0.110 b	0.135 d
SB 0.5 L	21.6	24.6 ab	236	254	194 a	165 b	5.78 b	4.18	3.74 ab	5.90	0.111 b	0.149 c
SB 1.0 L	22.6	25.1 a	240	256	192 a	163 bc	5.62 b	4.03	4.03 a	6.23	0.117 b	0.154 bc
SB 1.5 L	23.5	25.4 a	242	253	181 b	154 cd	5.80 b	3.93	4.05 a	6.45	0.130 a	0.166 a
SB 2.0 L	23.5	25.4 a	245	259	179 b	155 d	5.74 b	4.10	4.12 a	6.23	0.131 a	0.164 ab
*p*-value	0.1355	0.0756 *	0.1963	0.5988	0.0002 *	0.0035 *	0.0552 *	0.5568	0.0725 *	0.1426	0.0013 *	0.0019 *
C.V. (%)	6.09	3.52	4.94	4.40	2.03	4.13	4.33	5.63	8.52	6.33	5.53	5.67
	**14 DAA at the V_4_ Phenological Stage**											
Absolute Control	21.5	21.3 b	203	201	187	149	5.76	4.00	3.75 c	5.30 b	0.115 c	0.143
SB 0.5 L	22.1	22.6 ab	203	213	179	149	5.18	3.55	4.28 b	6.45 a	0.123 b	0.152
SB 1.0 L	23.1	22.9 a	207	207	175	146	5.36	3.78	4.34 b	6.08 a	0.133 a	0.159
SB 1.5 L	23.5	23.1 a	210	216	172	144	5.27	3.88	4.52 ab	6.00 a	0.137 a	0.161
SB 2.0 L	23.5	23.6 a	214	214	171	143	4.93	3.80	4.88 a	6.23 a	0.138 a	0.165
*p*-value	0.1128	0.0762 *	0.2361	0.2964	0.1844	0.6254	0.2311	0.4078	0.0072 *	0.0630 *	0.0003 *	0.1243
C.V. (%)	5.27	4.61	3.78	4.86	5.60	5.30	8.96	8.34	7.79	8.32	4.37	7.64
**Time B**												
	**7 DAB at the R_1_ Phenological Stage**											
Absolute Control	22.4 c	22.5 c	245	159	182	166	4.16	4.33	5.40	5.20 c	0.124 c	0.136
SB 0.5 L	23.0 bc	22.9 bc	245	161	176	165	3.94	4.23	5.87	5.45 bc	0.132 bc	0.140
SB 1.0 L	24.3 ab	23.7 ab	247	161	180	167	4.10	4.28	5.95	5.55 abc	0.136 bc	0.142
SB 1.5 L	25.5 a	23.9 ab	255	164	171	168	3.98	4.13	6.43	5.78 ab	0.150 a	0.143
SB 2.0 L	24.9 a	24.3 a	260	161	174	165	3.93	4.10	6.29	5.88 a	0.142 ab	0.148
*p*-value	0.0268 *	0.0799 *	0.4619	0.6787	0.4873	0.9247	0.6733	0.5148	0.1694	0.0555	0.0286 *	0.2482
C.V. (%)	5.17	3.76	5.60	3.05	5.33	3.60	6.43	4.93	9.69	5.43	7.15	4.82
	**14 DAB at the R_1_ Phenological Stage**											
Absolute Control	22.2 b	22.4 c	208 b	196	175	163 a	4.35	4.15	5.13	5.40 b	0.127	0.138 c
SB 0.5 L	23.3 ab	23.2 b	218 ab	202	172	159 ab	4.28	3.95	5.46	5.90 a	0.136	0.146 b
SB 1.0 L	24.2 a	23.8 ab	224 a	200	171	157 b	4.43	4.00	5.47	5.98 a	0.142	0.152 ab
SB 1.5 L	24.3 a	24.1 a	227 a	204	173	154 b	4.25	3.98	5.72	6.05 a	0.140	0.156 a
SB 2.0 L	24.0 a	24.4 a	226 a	203	174	154 b	4.15	4.00	5.80	6.13 a	0.138	0.159 a
*p*-value	0.0951 *	0.0017 *	0.0643 *	0.1958	0.9783	0.0512 *	0.4556	0.5606	0.1333	0.0305 *	0.3019	0.0014 *
C.V. (%)	4.54	2.29	4.13	2.43	5.34	2.66	4.90	4.42	6.46	4.95	7.29	3.71

* Data were analyzed by analysis of variance (ANOVA), and when significant differences were identified, means were compared using Fisher’s protected least significant difference (LSD) test at *p* < 0.10. ** Columns with different letters are significantly different according to the LSD test (*p* < 0.10).

**Table 3:** Contents of hydrogen peroxide (H_2_O_2_) and malondialdehyde (MDA) and activities of superoxide dismutase (SOD) and catalase (CAT) in soybean diagnostic leaves as a function of foliar SB application during the 2023/24 and 2024/25 growing seasons.

Treatment	H_2_O_2_		MDA		SOD		CAT	
	µmol g^−1^ Fresh Weight		nmol g^−1^ Fresh Weight		Units mg^−1^ Protein		µmol min^−1^ mg^−1^ Protein	
	2023/24	2024/25	2023/24	2024/25	2023/24	2024/25	2023/24	2024/25
**Time A**								
**7 DAA at the V_4_ Phenological Stage**								
Absolute Control	106 a **	215 a	20.3 a	22.3 a	163 a	149 a	7.73 a	7.13 a
SB 0.5 L	105 a	206 ab	19.1 ab	20.7 b	161 a	140 ab	7.54 ab	6.48 ab
SB 1.0 L	101 ab	196 bc	17.9 b	19.6 b	149 b	130 bc	6.74 c	6.08 b
SB 1.5 L	91.6 bc	188 c	14.7 c	17.7 c	150 b	128 c	7.03 bc	5.65 b
SB 2.0 L	87.7 c	190 c	14.8 c	17.4 c	147 b	130 bc	6.83 c	5.65 b
*p*-value	0.0468 *	0.0127 *	0.0003 *	0.0007 *	0.0147 *	0.0126 *	0.0293 *	0.0862 *
C.V. (%)	9.17	4.99	8.22	6.52	4.18	5.95	6.15	12.4
	**14 DAA at the V_4_ Phenological Stage**							
Absolute Control	124 a	182 a	20.6 ab	20.2 a	163	149 a	7.11	6.39
SB 0.5 L	115 ab	179 a	18.8 ab	19.5 a	159	144 ab	6.36	6.24
SB 1.0 L	119 a	175 a	20.8 a	19.2 a	162	139 bc	5.98	6.34
SB 1.5 L	104 bc	166 b	18.7 b	17.9 b	155	134 c	5.51	5.66
SB 2.0 L	101 c	166 b	15.2 c	17.4 b	158	133 c	5.51	5.51
*p*-value	0.0284 *	0.0082 *	0.0035 *	0.0083 *	0.2310	0.0208 *	0.1146	0.1482
C.V. (%)	8.73	3.57	8.97	5.24	3.46	4.46	14.4	9.58
**Time B**								
	**7 DAB at the R_1_ Phenological Stage**							
Absolute Control	202	191 a	24.1 a	24.2 a	158 a	160 a	7.53 a	8.72
SB 0.5 L	195	190 a	23.5 ab	23.1 a	153 ab	157 a	5.85 b	8.64
SB 1.0 L	196	183 ab	23.1 ab	23.1 a	151 bc	153 ab	5.87 b	7.91
SB 1.5 L	194	181 b	21.7 bc	22.3 a	147 c	148 b	5.64 b	7.88
SB 2.0 L	191	179 b	21.0 c	19.6 b	150 bc	145 b	5.83 b	7.83
*p*-value	0.2972	0.0726 *	0.0728 *	0.0427 *	0.0405 *	0.0708 *	0.0100 *	0.4027
C.V. (%)	3.29	3.53	6.66	8.37	2.80	4.76	10.9	10.4
	**14 DAB at the R_1_ Phenological Stage**							
Absolute Control	237	197 a	18.4 a	19.0 a	148 a	158	6.74 a	6.48
SB 0.5 L	233	192 a	18.3 a	18.0 a	146 a	152	5.43 b	5.85
SB 1.0 L	226	190 ab	17.9 a	14.9 b	143 ab	148	4.58 c	6.15
SB 1.5 L	231	183 bc	15.0 b	15.9 b	137 c	145	4.14 c	6.22
SB 2.0 L	227	178 c	15.5 b	15.7 b	140 bc	144	4.03 c	6.18
*p*-value	0.2352	0.0272 *	0.0041 *	0.0021 *	0.0182 *	0.1349	0.0003 *	0.7604
C.V. (%)	2.99	4.06	7.40	7.18	2.85	5.15	12.9	10.6

* Data were analyzed by analysis of variance (ANOVA), and when significant differences were identified, means were compared using Fisher’s protected least significant difference (LSD) test at *p* < 0.10. ** Columns with different letters are significantly different according to the LSD test (*p* < 0.10).

**Table 4:** Final plant population (FPP), root dry weight (RDW), plant height (PH), number of pods per plant (NPP), number of grains per pod (NGP), total number of grains per plant (TNGP), 100 grain weight (W100) and grain yield (GY) in soybean diagnostic leaves as a function of foliar SB application during the 2023/24 and 2024/25 growing seasons.

Treatment	FPP		RDW		PH		NPP		NGP		TNGP		W100		GY	
	ha^−1^ (×1000)		g Plant^−1^		cm		Numbers						g		kg ha^−1^	
	2023/24	2024/25	2023/24	2024/25	2023/24	2024/25	2023/24	2024/25	2023/24	2024/25	2023/24	2024/25	2023/24	2024/25	2023/24	2024/25
Absolute Control	254	239	4.13 b **	4.22	62.4	95.0 d	52.5	55.3	2.27	2.25 b	119 b	124 b	15.5	13.7	4434 c	4057 c
SB 0.5 L	254	239	4.28 ab	4.43	63.2	96.4 cd	53.4	55.3	2.34	2.25 b	125 ab	124 b	15.6	13.8	4685 b	4090 bc
SB 1.0 L	256	236	4.45 a	4.50	63.6	101 bc	54.9	58.3	2.30	2.31 a	126 ab	134 a	16.0	14.0	4916 a	4430 ab
SB 1.5 L	254	237	4.58 a	4.24	65.1	104 ab	57.1	58.2	2.32	2.34 a	133 a	136 a	15.6	13.9	4948 a	4462 a
SB 2.0 L	254	239	4.58 a	4.54	64.4	106 a	55.7	59.1	2.31	2.26 b	129 a	133 a	15.5	14.2	4782 ab	4521 a
*p*-value	0.9580	0.9778	0.0933 *	0.2887	0.7681	0.0130 *	0.2488	0.4995	0.2943	0.0095 *	0.0965 *	0.0666 *	0.2208	0.3077	0.0087 *	0.0607 *
C.V. (%)	1.47	4.34	5.60	5.66	4.85	4.18	5.32	6.64	1.98	1.61	4.95	5.28	2.27	2.62	3.68	5.87

* Data were analyzed by analysis of variance (ANOVA), and when significant differences were identified, means were compared using Fisher’s protected least significant difference (LSD) test at *p* < 0.10. ** Columns with different letters are significantly different according to the LSD test (*p* < 0.10).

**Table 5:** Soil classification and chemical characterization in the experimental area.

Soil Classification	Unit Chemical Properties	Botucatu—SP
		Typic Haplorthox
pH	CaCl_2_	5.8
Soil Organic Matter	g dm^−3^	24
Phosphorus	mg dm^−3^	31
Sulfur	mg dm^−3^	11
Aluminum	mmol_c_ dm^−3^	0
Potential Acidity	mmol_c_ dm^−3^	32
Potassium	mmol_c_ dm^−3^	3.9
Calcium	mmol_c_ dm^−3^	35
Magnesium	mmol_c_ dm^−3^	15
Sum of Bases—SB	mmol_c_ dm^−3^	53.9
Cation Ex. Capacity	mmol_c_ dm^−3^	85.9
Base Saturation	%	63
Aluminum Saturation	%	0
Iron	mg dm^−3^	22
Copper	mg dm^−3^	2.8
Manganese	mg dm^−3^	15
Zinc	mg dm^−3^	2.1
Boron	mg dm^−3^	0.39
Nickel	mg dm^−3^	0.5
Sand	g dm^−3^	117
Silt	g dm^−3^	281
Clay	g dm^−3^	502
Soil Density	g dm^−3^	1.19

**Table 6:** Experimental management timeline for the 2023/2024 and 2024/2025 seasons.

Management	Description	
	2023/2024	2024/2025
Production system	No-tillage	
Cultivar	NEO610 IPRO	
Row spacing	0.45 m	
Base fertilization	17.6 kg ha^−1^ N and 83.2 kg ha^−1^ P_2_O_5_	
Sowing	11 November 2023	16 November 2024
Emergence of seedlings	17 November 2023	23 November 2024
Topdressing fertilization	6 December 2023—90 kg ha^−1^ K_2_O	11 December 2024—90 kg ha^−1^ K_2_O
Application at V_4_ (Time A)	19 December 2023	12 December 2024
Analysis—7th day after application A (DAA)	26 December 2023	19 December 2024
Analysis—14th DAA	2 January 2024	26 December 2024
Application at R_1_ (Time B)	9 January 2024	4 January 2025
Analysis—7th day after application B (DAB)	16 January 2024	11 January 2025
Analysis—14th DAB	23 January 2024	18 January 2024
Harvest	20 March 2024	27 March 2024

**Table 7:** Treatment descriptions and application timing in the 2023/2024 and 2024/2025 seasons.

Treatments		Application		
		Dose (L ha^−1^)	Number	Timing
1	Control	only water	2	A; B
2	SB 0.5	0.50	2	A; B
3	SB 1.0	1.00	2	A; B
4	SB 1.5	1.50	2	A; B
5	SB 2.0	2.00	2	A; B

## Data Availability

The raw data supporting the conclusions of this article will be made available by the authors on request.

## Abbreviations

The following abbreviations are used in this manuscript:

ROS	Reactive oxygen species
RuBisCO	Ribulose-1,5-bisphophate carboxylase/oxygenase
7 DAA	7th day after application A (first application)
14 DAA	14th day after application A (first application)
7 DAB	7th day after application B (second application)
14 DAB	14th day after application B (second application)
Chl *a*	Chlorophyll a
Chl *b*	Chlorophyll b
Chl t	Total chlorophyll
Car t	Total carotenoids
C_S1_	First crop
C _S2_	Second crop
*A*	Net photosynthetic rate
gs	Stomatal conductance
Ci	Internal CO_2_ concentration
E	Foliar transpiration
WUE	Water use efficiency
CE	Carboxylation efficiency
H_2_O_2_	Hydrogen peroxide
MDA	Malondialdehyde
SOD	Superoxide dismutase
CAT	Catalase
FPP	Final plant population
RDW	Root dry weight
PH	Plant height
NPP	Number of pods per plant
NGP	Number of grains per pod
TNGP	Total number of grains per plant
W100	100 grain weight
GY	Grain yield
V_2_	Vegetative phenological stage—fourth fully developed trifoliate leaf
R_1_	Reproductive phenological stage—beginning of flowering
CO_2_	Carbon dioxide
RDC	Research and Dissemination Center
pH	Potential of hydrogen
Aw	Tropical savanna
RCBD	Randomized complete block design
N	Nitrogen
P_2_O_5_	Phosphorus pentoxide
K_2_O	Potassium oxide
DMF	N,N-dimethylformamide
PVPP	Polyvinylpolypyrrolidone
DTT	Dithiothreitol
EDTA	Ethylenediaminetetraacetic acid
TBA	Thiobarbituric acid
TPE	1,1,3,3-tetramethoxypropane
ANOVA	Analysis of variance
